# A call for immediate action to increase COVID-19 vaccination uptake to prepare for the third pandemic winter

**DOI:** 10.1038/s41467-022-34995-y

**Published:** 2022-12-06

**Authors:** Cornelia Betsch, Philipp Schmid, Pierre Verger, Stephan Lewandowsky, Anna Soveri, Ralph Hertwig, Angelo Fasce, Dawn Holford, Paul De Raeve, Arnaud Gagneur, Pia Vuolanto, Tiago Correia, Lara Tavoschi, Silvia Declich, Maurizio Marceca, Athena Linos, Pania Karnaki, Linda Karlsson, Amanda Garrison

**Affiliations:** 1 University of Erfurt, Erfurt, Germany; 2 Bernhard-Nocht-Institute for Tropical Medicine, Hamburg, Germany; 3 Faculty of Medicine, Southeastern Regional Health Observatory, Marseille, France; 4 School of Psychological Science, University of Bristol, Bristol, UK; 5 University of Turku, Turku, Finland; 6 Max Planck Institute for Human Development, Berlin, Germany; 7 University of Coimbra, Coimbra, Portugal; 8 European Federation of Nurses Associations, Brussels, Belgium; 9 Faculté de Médecine et des Sciences de la Santé, University of Sherbrooke, Sherbrooke, QC Canada; 10 Tampere University, Tampere, Finland; 11 Global Health and Tropical Medicine, GHTM, Instituto de Higiene e Medicina Tropical, IHMT, Universidade Nova de Lisboa, Lisbon, Portugal; 12 Department of Translational Research and New Technologies in Medicine and Surgery, University of Pisa, Pisa, Italy; 13 Istituto Superiore di Sanità, Italian National Institute of Health, Rome, Italy; 14 Department of Public Health and Infectious Diseases, Sapienza University of Rome, Rome, Italy; 15 Prolepsis Institute, Marousi, Athens Greece

**Keywords:** Preventive medicine, Interdisciplinary studies, Communication, Human behaviour

## Abstract

This Comment piece summarises current challenges regarding routine vaccine uptake in the context of the COVID-19 pandemic and provides recommendations on how to increase uptake. To implement these recommendations, the article points to evidence-based resources that can support health-care workers, policy makers and communicators.

COVID-19 vaccine uptake is suboptimal in many countries. In the European Union, for example, uptake is at 72%, with countries ranging between about 50% and 90% of the adult population being vaccinated twice. In North America, 65% are vaccinated twice, in lower middle income countries 56% are vaccinated twice. Immunity through infection or vaccination will have waned significantly by late 2022, and third and fourth booster shots are recommended. However, as of mid November, only 46% have received the booster in Europe (59% European Union), 42% in North America, 17% in lower middle income countries^1^. Governments in the northern hemisphere, where colder temperatures are likely to lead to winter COVID-19 waves, face the challenge of promoting vaccination uptake. This task includes encouraging people to access vaccination services, especially those in vulnerable and marginalised groups. Relatedly, it will also be crucial to reach pre-COVID-19 coverage for other recommended vaccinations—the World Health Organisation (WHO) and the United Nations International Children’s Emergency Fund (UNICEF) have sounded the alarm, acknowledging the largest backslide in routine vaccinations in three decades (WHO press release, July 15, 2022). Moreover, in Europe, the unprecedented pressure to protect refugees from Ukraine means that some countries will face additional challenges in maintaining functioning and reliable health-care systems. After living through almost 3 years of a pandemic, citizens are neither immunologically nor psychologically naïve. Therefore, future vaccination protocols and communication initiatives must account for the full range of individual experiences of vaccination and infection. Meanwhile, the impacts of past health policies must also be considered. In this article, we will summarise three major challenges before offering recommendations on how to meet them. We conclude by providing a number of resources to help implement the recommendations.

## Current challenges

One major challenge is the fact that among still unvaccinated adults, most are unwilling to get vaccinated; meanwhile, many children remain unvaccinated because of their caretakers’ reluctance^2^. In some countries, despite great efforts to improve access and motivation, many vulnerable individuals have yet to get vaccinated (e.g. older people, people living in deprived areas, migrants, people without housing, people living in prison). Major barriers to vaccine uptake include doubts regarding vaccine safety or effectiveness, a lack of trust in authorities^3^, and administrative and logistical difficulties^4^. These issues can be exacerbated by exposure to misinformation, information overload or conflicting information^5,6^.

A second challenge is that individuals increasingly have personal experiences with the virus and vaccination^7^, which may lower their willingness to get vaccinated. Many who were previously infected will have experienced COVID-19 as a non-severe illness and may therefore feel no need to get vaccinated after recovery^8^. However, current evidence suggests that receiving at least one vaccination dose after a SARS-CoV2 infection significantly reduces the risk of reinfection^9^. Among the vaccinated majority, adverse health-related events that follow soon after vaccination may be attributed to the vaccination^10^ even though temporal contiguity does not, by itself, imply causality. Media discussions about a ‘post-vac’ syndrome and the extent to which adverse events are fully reported may also raise doubts about the safety of vaccines. These questions can discourage the receipt of additional doses^11^, particularly when individuals assume that they have acquired some immunity.

Third, policy changes may undermine the perceived importance of vaccinations. For example, some countries, e.g. Germany, have discussed mandatory regulations but then did not pass them^12^. Other countries, such as Austria, discarded mandatory policies related to COVID-19 before implementing them^12^. France, for example, included the booster vaccine in the green pass and abandoned the green pass only shortly after that. Meanwhile, authorities often allowed regulations to expire or did not enforce policies. In Germany, for instance, the mandatory vaccination regulation for health-care professionals was loosened in some federal states which may partially be due to a lack of health-care personnel. Such changes likely sent conflicting messages about the necessity and benefits of vaccination—both to the public and to health workers, the group most frequently targeted by mandatory regulations.

## Recommendations to meet the challenge

A crowd-sourcing study made use of many health experts and explored a large number of possible interventions to increase booster vaccine uptake^13^. Two of the five interventions rated as both effective and acceptable by experts and members of the general public related to health-care personnel engagement—mobile vaccination teams and promotional campaigns. Three interventions were structural, such as providing incentives (e.g. day off, money, tax benefits). These structural interventions indicate that effective behavioural science mechanisms should be used to improve health-care systems and vaccination programmes. Other studies have shown that trust is a major factor impacting people’s willingness to get vaccinated^3^. Research also highlights the importance of people’s confidence in vaccine safety; adverse reactions to previous shots might limit the uptake of boosters^11^. Based on the existing studies and the challenges raised above, it is clear that patients’ concerns need to be carefully addressed. In addition, the health-care system should make services easily accessible. Health-care personnel (HCP) is key for increasing vaccine uptake, as they connect the patient to the health system.

HCP are also people—their own hesitancy may make them less likely to recommend vaccination^14^. Meanwhile, talking to sceptical patients may be a personal challenge. As a most trusted source of information, HCP need support in their everyday work. For example, if they are to engage effectively with hesitant patients, debunk misinformation and encourage vaccination, they need resources that help them address misinformation and find the right tone for motivating patients. They might also benefit from additional competencies for involving vulnerable and marginalised individuals. While misperceptions about COVID-19 can indeed hinder vaccination intentions^15^, there is a body of research indicating that communication alone will not remedy the problem^16,17^. Indeed, evidence about “what works” is mixed^18^. From a behavioural science perspective, there are two complementary building blocks that can increase vaccine uptake: (1) reducing psychological frictions to enable vaccination behaviour, which can be achieved by optimising the health system—and (2) improving communication measures to tackle vaccine hesitancy and misinformation.

To address these challenges, five current EU-funded multisectoral, multidisciplinary and international research projects (detailed in the funding section) are developing evidence-based strategies to overcome vaccine hesitancy and structural barriers to vaccination in the European region. As researchers involved in these projects, we jointly advance the following four recommendations. We urge governments and relevant stakeholders—politicians, health authorities, communities and health communicators—to act now to support HCP and vaccination programmes in preparing for the third pandemic winter. Because routine vaccinations have diminished over the course of the pandemic, too, it is also crucial to increase uptake of recommended vaccines at all ages—and HCP play a major role in this process as well.

As a first recommendation, we suggest to strengthen the health system by implementing lessons learnt from behavioural science. Second, tailored approaches should be developed to reach the vulnerable and underserved groups using systematic listening activities. Third, evidence-based resources should be provided to support health-care workers in working with hesitant patients. Fourth, it is necessary to engage with the media.

These four recommendations will be detailed in the subsequent sections of this article, with additional resources for implementation following.

1. **Strengthen the health system by implementing lessons learnt from behavioural science.**
  Improving the health-care system infrastructure could ease navigation and facilitate access to and use of vaccination services. Such improvements could, for example, entail a higher level of digitalisation (e.g. vaccination registers, digital health records, and/or text message systems enabling direct messaging to vulnerable groups). Research shows that receiving invitations to appointments^19–21^, links via text messages for appointment booking^16^ and text message reminders before regular appointments^22^ leads to increased uptake. Mobile vaccination teams can help facilitate access^13^, and mandatory regulations may be appropriate under certain conditions^23^. Generally, making vaccination an easy choice for everyone should be the goal^24^. Strengthening the health-care system means identifying barriers—ranging from human laziness, procrastination, forgetfulness and complacency to actual difficulties accessing services—and ensuring easy and equitable access by removing administrative, logistical and other practical and psychological barriers, especially for underserved groups. Even if some of the aforementioned systems-level changes may have mid- or long-term effects only, it would be worth the investment to address the weaknesses that the pandemic has revealed, especially given the backdrop of declining childhood immunisation rates^25^.
2. **Develop tailored approaches using systematic listening activities.**
  In most countries where vaccines were widely available, the majority of citizens understand the importance of vaccination against COVID-19 and have willingly complied with the established protocols^26^. Nevertheless, it is important to provide ongoing support for this majority. Sustained evidence-based and tailored communication is crucial^27^, and such communication should actively address the questions that people are asking: Am I eligible for another dose? Should I get another dose now or in the winter? Can vaccination protect against long COVID? Why do so many people get infected despite vaccination? Will I need another dose despite previous vaccination(s) and infection? What about vaccines against the new variants? Communication efforts should refer explicitly to the high level of protection that vaccines provide against severe disease and death while managing expectations regarding the much briefer protection against infection or transmission^9^. Side effects and adverse events following immunisation should also be communicated transparently to maintain trust and counter misinformation^28^.
  Communication efforts should also focus on vulnerable groups to improve equity in vaccine access and uptake. Such efforts would benefit from identifying segments of the population and reaching out to them using tailored approaches^29^. Developing such strategies involves listening to the public or special target groups by conducting qualitative and quantitative research to understand how they can be reached while addressing underlying reasons for hesitancy^30^. Appropriate communication measures should be action-oriented (who should do what, when, why and how), and they should be designed with mindfulness around the circumstances of these groups and their different experiences with COVID-19. These measures should include providing and updating readily available and trustworthy sources of information about COVID-19, transparently explaining the risks of long COVID describing the benefits and risks of vaccination and addressing COVID-19 and vaccination-related myths, and of scientific uncertainties in the context of COVID-19^31^. Countries should invest in easy-to-follow and transparent health communication for all languages and literacy levels while ensuring that HCP and the general public know about these offerings. Where possible, it would be advisable to deploy trusted community-based vaccination champions who are willing to engage in dialogue and support communication activities^32,33^.
3. **Provide evidence-based resources to support health-care personnel.**
  Action should be taken to monitor the challenges faced by HCP when interacting with the public and with patients during vaccination campaigns. HCP should have special priority in these campaigns. HCP should be made aware of reliable sources of vaccination information that they can share with patients. Misinformation about vaccines is rampant and has demonstrable adverse effects on vaccination intentions^15,34,35^. It is often necessary to address misinformation and misconceptions, which may make patient–provider conversations difficult. Therefore, HCP should be offered education and training on relevant evidence-based communication interventions and techniques to address vaccine hesitancy and debunk misinformation. HCP should be encouraged and supported in their engagement with people exhibiting low health literacy as well as with people of different cultural backgrounds. To strengthen trust, HCP need to engage in transparent and empathic communication that takes concerns seriously while responding to questions regarding vaccine safety; doing so without hiding the negative features of vaccines can also strengthen trust^28^, one of the core factors underlying high vaccine uptake^3,36^.
4. **Engage with the media.**
  Finally, engaging with and supporting the media is recommended not only for governments, but also for National Public Health Institutes. Social media platforms can be leveraged to inform different audiences about the benefits and risks of vaccination; they may also be used to communicate the consensus reached by doctors and the medical community^37^. Such communication increases uptake, as demonstrated by a field study in the Czech Republic^37^. However, it is also necessary to engage with and train (science) journalists and other media personnel to report effectively and correctly regarding health emergencies while avoiding the risks of a false balance in media coverage^38–40^. Moreover, investing in training HCP on how to face vocal vaccine deniers in public debates can pay off. Research shows that being able to recognise and demask the deniers’ strategies protects audience members attending debates^34,40^.

## Resources

Scientists and health organizations have developed a large number of evidence-based resources for use by practitioners who can support the required actions. The remainder of this article will describe a number of resources that can facilitate the implementation of the above recommendations. Table 1 contains links and evidence supporting the effectiveness of these resources.

**Table 1:** Overview of resources: Descriptions of resources in the text

Resource	Evidence base
WHO guidance for tailoring immunisation programmes (TIP)	
https://www.euro.who.int/en/health-topics/disease-prevention/vaccines-and-immunisation/publications/2019/tip-tailoring-immunisation-programmes-2019	The resource is grounded in evidence which based on experiences in 12 countries within and outside the European region from 2013 to 2019. An external committee of six global experts conducted a review in 2016 informed by country assessments, a review of national and regional documents, and an online regional survey. Various publications document the successful application of TIP^42–45^.
COVID-19 vaccination communication handbook	
https://sks.to/c19vax	The handbook was created by a team of academics from diverse disciplines, drawing on decades of research in their respective fields. It summarises and highlights the relevant facts and recommendations regarding vaccine communication in the context of COVID-19. The handbook relies on guides and documents from health organisations, public bodies and researchers, as well as published scientific research on vaccine hesitancy, science communication and debunking misinformation. The handbook provides detailed references to evidence for each of the recommendations it makes.
Motivational-interviewing training materials	
https://apps.who.int/iris/handle/10665/340751	Studies in Canada, including multicentre randomised controlled trials^47,49,56^, have proven the effectiveness of the motivational-interviewing approach^50^. Both vaccine hesitancy and uptake were positively affected by these interventions. Since 2018, the PromoVac strategy, an educational intervention based on the motivational-interviewing approach, has been successfully implemented as a new practice of care in maternity wards across the province of Quebec through the Entretien Motivationnel en Maternité pour l’Immunisation des Enfants (EMMIE) programme.
https://www.cdc.gov/vaccines/covid-19/hcp/engaging-patients.html	
https://psychwire.com/motivational-interviewing/addressing-vaccine-hesitancy	
https://www.canvax.ca/canvax-webinar-series	
Educational resources for health-care personnel to help address vaccine misconceptions	
https://jitsuvax.info/	The resource is based on a systematic literature review of 152 scientific articles, a thematic analysis of 2066 anti-vaccination arguments, which identified 11 underlying attitude roots (i.e. psychological constructs underlying people’s deeply-held beliefs) and more than 60 anti-vaccination “memes” that represent common instantiations of those roots. The resource guides the user through rebuttals of those memes that are cognisant of the underlying attitude root and provide affirmation of the patient’s deeply-held beliefs while also correcting a particular meme.
Handbook on how to debunk misinformation	
https://skepticalscience.com/debunking-handbook-2020-downloads-translations.html	The Debunking-Handbook 2020^57^ is based on an expert review of 22 experts whose research focuses on countering misinformation (selection process of experts: https://www.climatechangecommunication.org/wp-content/uploads/2020/10/DB2020paper.pdf). Studies have shown that debunking or prebunking misinformation by using a fact-sandwich (Table 2)^35^ or other optimised forms of refutation messages^58^ successfully reduces the impact of misinformation, at least in the short term. Moreover, meta-analyses on debunking show that correcting misinformation in social media posts works^59,60^.
How to respond to vocal vaccine deniers in public	
https://apps.who.int/iris/handle/10665/343301	The central recommendations of the WHO guidance documents on how to rebut misinformation were tested in nine experimental studies across two peer-reviewed research articles, showing that rebutting misinformation either by uncovering the rhetorical techniques of science denialism or by stating the scientific facts (or both) reduces the negative effects of the vaccine denier^34,40^.

### To implement recommendations 1 and 2

The Tailoring Immunisation Programmes (TIP) approach was developed by the WHO/Europe to support countries in identifying “populations with suboptimal vaccination uptake; barriers to and drivers of vaccination in those population groups; [and] interventions to address barriers and leverage drivers of vaccination—with the aim of increasing vaccination uptake”^14^. Its step-by-step methodology is laid out in a comprehensive workbook and can be used as a diagnostic instrument^41^ to guide informed action, such as improving behaviour management and tailoring communication activities. It has been successfully used in 12 countries (e.g.^42–45^). In Bosnia and Herzegovina, for example, interviews with HCP identified drivers and barriers for vaccination^46^ and informed subsequent policy recommendations. TIP currently also serves as the basis for the EU-funded VAX-TRUST project, which designs and implements interventions for health-care professionals in seven European countries.

### To implement recommendations 1–4

The COVID-19 Vaccination Communication Handbook serves governments, policy makers, practitioners, journalists and the public. It includes two related products: (i) A PDF handbook of step-by-step recommendations specifically about COVID-19 vaccines, which summarises the principal factors that drive vaccine uptake in general, how these factors can be applied to the case of COVID-19, what to expect from and how to respond to vaccine opponents, and how specific misinformation relating to the vaccine can be prebunked or debunked. This handbook has been translated into 12 different languages to date. (ii) A crowd-sourced website where experts provide evidence-based advice on how to communicate effectively about COVID-19 vaccines from different perspectives (e.g. communications, behaviour, public health, incentives, personality and beliefs). The information has been updated based on recent and emerging evidence about COVID-19 and vaccines.

### To implement recommendation 3

Because HCP are one of the most important sources of information for patients, efforts should be made to improve interpersonal vaccine communication. Several trials have demonstrated that motivational-interviewing can significantly reduce vaccine hesitancy and increase vaccine uptake^14,47–49^. Motivational-interviewing involves guiding patients towards change by expressing empathy and respecting individual autonomy^50,51^. Understanding person-specific drivers of hesitancy allows clinicians to efficiently provide tailored, accurate information that motivates individuals and reinforces confidence in their decisions. Motivational interviewing involves four steps: (i) engaging to establish a trustful relationship promoting safety to freely express opinions, beliefs and knowledge gaps; (ii) understanding what matters most to the individual; (iii) offering information to co-build accurate knowledge and guide the individual towards vaccine intention; and (iv) clarifying and accepting decisions to validate the individual’s decision-making autonomy. The ultimate goal is to build trust between the patient and the provider.

### To implement recommendation 3

The www.jitsuvax.info website developed by the EU-funded JITSUVAX project focuses on supporting HCP. The website seeks to equip HCP with psychological tools to go beyond addressing flaws in faulty arguments. It also helps them to consider the attitudinal roots of opposition to vaccines^52^—i.e. the underlying psychological attributes driving a person’s beliefs, such as moral or religious concerns or distrust. Understanding these underlying rationales can help HCP understand and address anti-vaccination arguments in face-to-face dialogues with patients^53–55^. The learning resource provides information about over 60 recurrent themes of contrarian arguments related to a broad range of attitudinal roots, with practical hands-on examples of how to affirm patients and correct their misconceptions while using empathy. These techniques can be combined with motivational interviewing or other dialogue tools and used as a training resource for HCP.

### To implement recommendations 3 and 4

The Debunking-Handbook 2020 provides guidance on when and how to properly debunk misinformation. Based on an extensive synthesis of evidence, it was written for engaged citizens, policy makers, journalists and other stakeholders. The handbook summarises the key ingredients of effective debunking (e.g. the fact-sandwich as shown in Table 2), which can guide the design of social media posts, press releases, news articles, health campaign flyers and website texts. This handbook is currently available in 14 different languages.

**Table 2:** Example of a fact-sandwich to debunk misinformation

Provide fact	FACT: COVID-19 vaccines have no impact on your fertility
Name myth	MYTH: “Being vaccinated could make me infertile.”
Explain fallacy	The myth that a COVID-19 vaccine could cause infertility arose on a blog with a long history of promoting conspiracy theories and misinformation. It is based on the idea that the vaccine works by triggering an immune response to a spike protein on the surface of the coronavirus. It is correct that a spike protein helps the virus enter cells, and it is also one of the ways the human body recognises a virus and knows to let its immune cells attack it^61,62^. However, this has nothing to do with fertility. This myth is based on the fact that there is overlap between a few components of the spike protein in the virus and in a placental protein. However, the overlap is too small to plausibly affect fertility. If the virus were related to fertility, COVID-19 would have to affect pregnancy outcomes, but this has not been observed^63–66^.
Provide fact again	The guidance published by the Association of Reproductive and Clinical Scientists and the British Fertility Society confirms that there is absolutely no evidence, and no theoretical reason, that any of the vaccines can affect the fertility of women or men^67^.

Example taken from the COVID-19 vaccination communication handbook, following advice detailed in the Handbook on How to Debunk Misinformation.

### To implement recommendation 4

Facing a vocal vaccine opponent is a challenge—especially when others are listening, and the discussion might be unsettling. The How to Respond to Vocal Vaccine Deniers in Public guidance document from the WHO’s Regional Office for Europe supports people in this very situation. This document can assist politicians or representatives of health organisations on talk shows or on social media sites as well as engaged citizens having discussions with friends or family. An evidence-informed framework helps in designing effective counter-messages against vaccine misinformation and introduces two strategies for effective rebuttal: uncovering common rhetorical flaws in anti-vaccination messages and countering vaccine misinformation by providing support for the scientific perspective. The central recommendations of this document include (1) avoiding giving centre stage to the denier and (2) rebutting misinformation either by uncovering rhetorical techniques of science denialism or by stating scientific facts. For example, if a vaccine denier states that “vaccines should be 100% safe”, the counterargument might point out that this statement is based on an impossible expectation: no medical product is ever 100% safe (i.e. technique rebuttal). In stating scientific facts, the rebuttal might highlight the high safety profile of vaccines in general (i.e. fact rebuttal). The effectiveness of these strategies was supported in a series of randomised controlled trials^34,40^.

## Use behavioural science to overcome the vaccination gap

In sum, there are enormous challenges to increasing the uptake of COVID-19 (booster) vaccines. Nonetheless, research from social and behavioural science can help provide an analysis of the situation and identify target groups and necessary interventions, both in terms of enabling behaviour and communication. The past decade has provided valuable tools grounded in extensive research and experience. While there is no guarantee that these strategies will work in all contexts, the presented resources are available at the current time and based on evidence from labs or the field and input from experts in the field. We urge researchers to use and further evaluate the tools, so that the evidence base can grow. Action is immediately required to help societies cope with the uncertain COVID-19-related challenges that lie ahead of us.
